# Cow’s milk allergy: evidence-based diagnosis and management for the practitioner

**DOI:** 10.1007/s00431-014-2422-3

**Published:** 2014-09-26

**Authors:** Carlos Lifschitz, Hania Szajewska

**Affiliations:** 1 Hospital Italiano de Buenos Aires, Buenos Aires, Argentina; 2 Department of Paediatrics, The Medical University of Warsaw, 01184 Warsaw, Działdowska 1, Poland

**Keywords:** Allergy, Children, Infants, Pediatrics

## Abstract

This review summarizes current evidence and recommendations regarding cow’s milk allergy (CMA), the most common food allergy in young children, for the primary and secondary care providers. The diagnostic approach includes performing a medical history, physical examination, diagnostic elimination diets, skin prick tests, specific IgE measurements, and oral food challenges. Strict avoidance of the offending allergen is the only therapeutic option. Oral immunotherapy is being studied, but it is not yet recommended for routine clinical practice. For primary prevention of allergy, exclusive breastfeeding for at least 4 months and up to 6 months is desirable. Infants with a documented hereditary risk of allergy (i.e., an affected parent and/or sibling) who cannot be breastfed exclusively should receive a formula with confirmed reduced allergenicity, i.e., a partially or extensively hydrolyzed formula, as a means of preventing allergic reactions, primarily atopic dermatitis. Avoidance or delayed introduction of solid foods beyond 4–6 months for allergy prevention is not recommended.

*Conclusion*: For all of those involved in taking care of children’s health, it is important to understand the multifaceted aspects of CMA, such as its epidemiology, presentation, diagnosis, and dietary management, as well as its primary prevention.

## Introduction

Cow’s milk allergy (CMA) is a common diagnosis in infants and children. It is clearly overdiagnosed in many cases, but it is also underdiagnosed in many others. Many health care professionals and parents alike confuse, at times, CMA with lactose malabsorption. Inappropriate elimination diets have been imposed on pregnant and lactating women and their infants to prevent allergies without scientific evidence proving their efficacy. Even when well indicated in infants and children diagnosed with an allergy, the type of dietary products to eliminate and the duration of such elimination are not always logical. Elimination of all cow's milk products, without appropriate substitutions, can lead to malnutrition and/or specific nutrient deficiencies at a time when infants and children are growing. For all of those involved in taking care of children’s health, it is important to understand the multifaceted aspects of CMA, such as its epidemiology, presentation, diagnosis, and dietary management, as well as its primary prevention. Recommended therapeutic modalities should be based on evidence. This is possible whenever enough studies in one particular area, in homogenous populations, help prove or disprove a certain diagnostic or therapeutic approach.

Here, we discuss current evidence and recommendations on the prevalence, natural history, clinical manifestations, diagnosis, management, and prevention of CMA aimed at the primary and secondary care providers. For this, MEDLINE was searched in May 2014. Preference was given to evidence and recommendations from scientific societies published in the last 4 years (2010–2014). Documents found to relate to both food allergy in children in general and CMA in particular were included. Among the documents found regarding general allergy are those by the US National Institute of Allergy and Infectious Diseases (NIAID 2010) [7] and International Collaboration in Asthma, Allergy and Immunology (International Consensus ON, ICON 2012) [9]. Among those specifically related to CMA are the ones published by the World Allergy Organization (WAO 2010) [18], the European Society for Paediatric Gastroenterology, Hepatology and Nutrition (ESPGHAN 2012) [31], and the British Society for Allergy and Clinical Immunology (BSACI 2014) [36]. Although in this review, we will present the available evidence, at the end, we will make some comments regarding areas of doubt that may occur in clinical practice.

## Definition

The topic of definition still causes confusion among physicians. Words such as “allergy,” “intolerance,” and “hypersensitivity” are used interchangeably. The accepted definition of allergy is “a hypersensitivity reaction triggered by specific immunologic mechanisms” [7, 28, 29] There is no such thing as “allergy to lactose” but rather lactose intolerance.

## Prevalence

Conclusions from a 2010 systematic review concluded that the evidence for the prevalence of food allergy is greatly limited by a lack of uniformity of the criteria for making a diagnosis. Consequently, it remains unclear whether the prevalence is increasing [10], although some data suggest it [9]. The prevalence of CMA in children living in the developed world is approximately 2 to 3 % [25, 56], making it the most common cause of food allergy in the pediatric population. Only among breastfed infants is the prevalence lower (0.5 %) [25]. These numbers most likely refer to IgE-mediated CMA, while the prevalence of non-IgE-mediated CMA is not well known.

## Principal allergens

The major cow’s allergens belong to the casein fraction of proteins (αs1-, αs2-, β-, and κ-casein) and to whey proteins (α-lactalbumin and β-lactoglobulin) [62]. There is some cross-reactivity with soy protein, particularly in non-IgE-mediated allergy. There are immune and non-immune-mediated allergic phenomena. Immune-mediated adverse food reactions can be classified into four major categories: IgE-mediated, non-IgE-mediated, mixed, and cell-mediated reactions [7]. CMA is most frequently caused by a non-IgE-mediated mechanism.

## IgE and non-IgE-mediated allergy

Two basic mechanisms explain allergic reactions to cow’s milk as well as to other food allergens: those mediated by IgE and those not mediated by IgE. The most common IgE-mediated manifestations of CMA are acute urticaria and angioedema. The most common non-IgE-mediated manifestations of CMA are involving skin and the gastrointestinal tract. At the level of the gastrointestinal tract, presentations include the following: (1) CM-induced enterocolitis syndrome which involves the entire gastrointestinal tract, (2) CM-induced enteropathy that involves only the small bowel, and (3) CM-induced proctitis and proctocolitis, involving the rectum and colon [18].

## Clinical manifestations

CMA is mostly a disease of infancy and early childhood. Affected infants present usually within the first 6 months of life, and one review reported that the majority of infants develop symptoms before 1 month of age, often within 1 week after the introduction of cow’s milk proteins to their diet [25]. However, breastfed infants can also be affected by dairy products ingested by the mother and eliminated in her breast milk. Rare is the onset of symptoms after 12 months of age [36]. The majority of affected children have one or more symptoms involving one or more organ systems, mainly the gastrointestinal tract and/or skin. One recent review suggests that gastrointestinal food allergies are commonly associated with a wide range of extra-intestinal manifestations such as fatigue, allergic shiners, mouth ulcers, joint pain/hypermobility, poor sleep, night sweats, headache, and bed wetting [13].

Symptoms of non-IgE-mediated CMA are mostly delayed reactions that occur beyond 2 h following ingestion) and usually involve the gastrointestinal tract and/or skin [54]. Symptoms such as urticaria and/or angioedema with vomiting and/or wheezing are suggestive of IgE-mediated CMA, which generally occur within minutes and up to 2 h of cow’s milk protein ingestion. The skin is frequently involved followed by the gastrointestinal tract and, least frequently, the respiratory and/or cardiovascular systems. The majority of reactions are mild to moderate, but life-threatening anaphylaxis (1–2 %) can also occur [36, 53] (Table 1). Together with peanuts and tree nuts, cow’s milk is one of the most common foods capable of causing anaphylactic reactions [27]. Evidence of sensitization (presence of specific IgE) is typical [53].

**Table 1:** Main characteristics of IgE-mediated and non-IgE-mediated allergy

Characteristic	IgE-mediated	Non-IgE-mediated
Time of exposure to reaction	Minutes to 2 h	Several hours to days
Severity	Mild to anaphylaxis	Mild to moderate
Duration	May persist beyond 1 year of age	Usually resolved by 1 year
Diagnosis	Specific serum IgE, skin prick tests	Oral challenge

Other IgE-mediated disorders include food protein-induced enterocolitis syndrome (the entire gastrointestinal tract is involved), food protein-induced enteropathy (small bowel), food protein-induced proctitis and proctocolitis (rectum and colon), and food-induced pulmonary hemosiderosis (Heiner’s syndrome) [53]. Mixed IgE- and non-IgE-mediated reactions, involving humoral and/or cell-mediated mechanisms, also manifest themselves at the level of the skin and/or gastrointestinal tract. Such entities include allergic eosinophilic gastrointestinal disorders and atopic dermatitis (eczema).

CMA is generally outgrown during early childhood or, at the latest, in adolescence. Overall, the chances of outgrowing an allergy are better for non-IgE-mediated CMA. Children at risk of not resolving the problem are those affected with IgE-mediated CMA who have high levels of milk-specific IgE antibodies, multiple food allergies, and/or concomitant asthma and allergic rhinitis. Such children are more likely to have a more prolonged persistence of sensitization [36, 51]. Greater chances of developing tolerance to cow’s milk were found in children with low levels of IgE binding to cow’s milk and specific IgE binding to α-lactalbumin, β-lactoglobulin, κ-casein, and αs1-casein [1]. Resolution of CMA within the first 5 years of life could be predicted by milk-specific IgE levels, skin prick test results, and the severity of atopic dermatitis [63]. A web-based calculator to determine the prognosis of children with CMA is available at www.cofargroup.org. Validation studies are still needed [36].

## Diagnosis

**Fig. 1:**
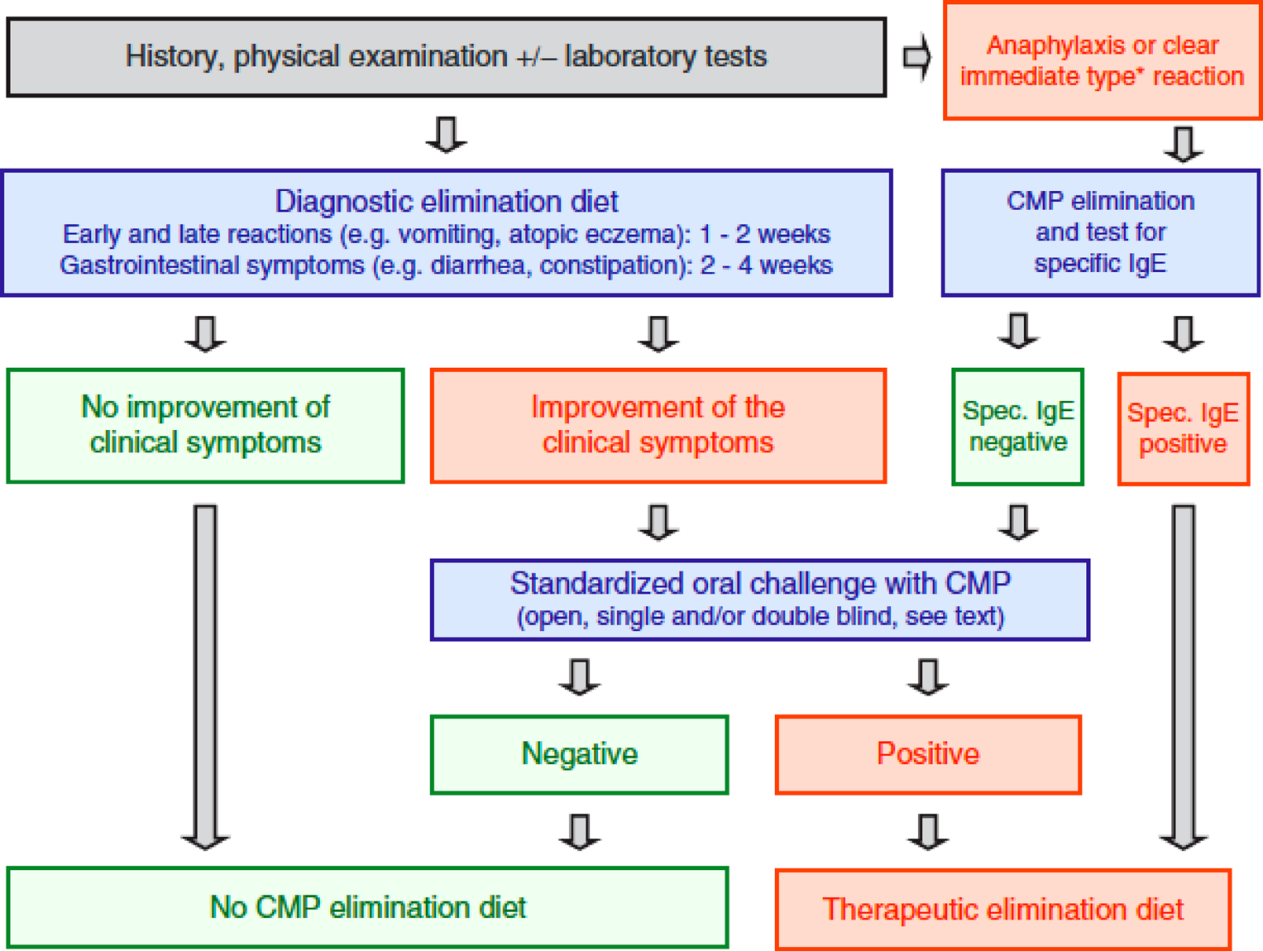
Evaluation of infants suspected of having cow’s milk protein allergy (CMP) according to the ESPGHAN criteria (Koletzko et al. [31], permission obtained). *eHF* extensively hydrolyzed formula *AAF* amino acid-based formula

Among other organizations, ESPGHAN has developed an algorithm for the evaluation of infants and children with symptoms compatible with the diagnosis of CMA (Fig. 1). In addition to the detailed medical history and physical examination, diagnostic elimination diets, skin prick tests (SPTs), specific IgE (sIgE) measurements, and oral food challenges are part of the routine work-up [7, 18, 31, 53]. If the patient is in the appropriate age range and the history and symptoms are consistent with the diagnosis of CMA, an open or single-blind challenge is often sufficient to make the diagnosis. However, a double-blind, placebo-controlled oral food challenge is still the gold standard for the diagnosis of food allergy [50]. When cow’s milk protein is the only suspected allergen, the diagnosis is simpler than on cases where the child is already ingesting a variety of foods. When multiple food allergies are suspected, published standards for office-based oral food challenges [40], as well as for double-blind, placebo-controlled, oral food challenges, should be followed [49].

A systematic review published recently looked into the specificity and sensitivity of tests employed for the diagnosis of food allergy. Results indicate that the existing evidence regarding the accuracy of such tests is limited, making their interpretation problematic [58]. In IgE-mediated food allergy, determination of SPT and sIgE seems to be sensitive, albeit not specific. For the specific case of IgE-mediated CMA, the values are as follows: sIgE, sensitivity 87 % (75 to 94) and specificity 48 % (36 to 59); SPT, sensitivity 88 % (76 to 94) and specificity 68 % (56 to 77). In non-IgE-mediated CMA, the value of tests is far more limited, and the clinician needs to rely on history, physical examination, and the results of the elimination diet and relapse upon milk challenge. The offending food challenge is the diagnostic gold standard [36]. Screening tests, such as SPTs, sIgE tests, and atopy patch tests, have been shown to lack specificity and sensitivity [7]. On the other hand, tests such as Vega (electrodermal), or cytotoxicity, iridology, kinesiology, food-specific IgG, pulse, and hair analysis are not recommended for the diagnosis of allergy because of the lack of any scientific evidence and reliability and reproducibility [9, 36]. Fecal studies for food particles or immune components are not reliable either.

## Management of cow’s milk allergy

Avoidance of cow’s milk protein in any form is the only available treatment [14, 21, 36]. In the case of breastfed infants, the mother should eliminate all dairy products from her own diet. It has to be considered that it may take up to 72 h to clear breast milk antigens ingested by the lactating woman. Calcium supplements should be added to the mother’s diet to replace milk intake [18, 31, 36]. From the practical standpoint, treatment of CMA imposes less sacrifices on the mother if the child was not being breastfed, provided that the family is given access or can afford the cost of special infant formulas. For infants 6 months old or younger, the recommended formulas for treatment of CMA are extensively hydrolyzed protein or amino acid-based formula. In infants older than 6 months, soy formula could be tried particularly in IgE-mediated cases.

## Which formula and to whom

### Extensively hydrolyzed formulas

The American Academy of Pediatrics defines as “extensively hydrolyzed formula” those containing only oligopeptides that have a molecular weight <3,000 Da to which at least 90 % of infants do not manifest any clinical symptoms in controlled double-blind studies [21]. Initial exclusive feeding of an extensively hydrolyzed formula is the treatment of choice for infants suspected of having CMA suffering from mild to moderate disease (see below). Because of its taste, some children may refuse to take the needed quantities for growth in which case an amino acid-based formula may be needed as their organoleptic characteristics are not as unpleasant.

### Amino acid formulas

These formulas, as the name indicates, provide protein only in the form of free amino acids and no peptides. Although in theory, amino acid formulas may be used as first-line treatment for CMA, their high cost may be a limiting factor. BSACI recommendations [36] for amino acid formulas include the following: infants and children with (1) severe CMA (failure to thrive and abundant blood in stools), (2) multiple food allergies, (3) allergic symptoms or severe atopic eczema when exclusively breastfed, (4) severe forms of non-IgE-mediated CMA, such as eosinophilic esophagitis, enteropathies, and food protein-induced enterocolitis syndrome, (5) growth faltering, and/or (6) infants at nutritional risk with reactions to or refusal to ingest appropriate amounts of extensively hydrolyzed formula.

### Amino acid formula vs. extensively hydrolyzed whey or casein formula

An amino acid rather than an extensively hydrolyzed formula is recommended for infants with IgE-mediated CMA at high risk of anaphylactic reactions (prior history of anaphylaxis and currently not receiving extensively hydrolyzed protein formula). Extensively hydrolyzed protein formula rather than an amino acid formula is recommended for infants with IgE-mediated CMA at low risk of anaphylactic reactions (no prior history of anaphylaxis or currently receiving an extensively hydrolyzed protein formula) [31, 36].

### Soy protein formula

The North American Society for Pediatric Gastroenterology, Hepatology and Nutrition and ESPGHAN recommendations agree that soy formulas should not be used in infants with food allergy under 6 months of age. Because of their lower cost and better palatability than extensively hydrolyzed formulas, soy protein formulas could be considered for use in patients with food allergy older than 6 months of age. In such cases, however, tolerance to soy protein should first be established by clinical challenge. Infants with IgE-mediated CMA allergy are more likely to tolerate soy formula than those with non-IgE-mediated CMA [6, 14].

### Extensively hydrolyzed whey or casein formula vs. soy formula

Use of extensively hydrolyzed milk formula rather than soy formula is recommended in infants with IgE-mediated CMA. Soy formula is not recommended in infants under 6 months of age [6, 14].

### Extensively hydrolyzed whey or casein formula vs. extensively hydrolyzed rice formula

Although tolerance and safety of an extensively hydrolyzed rice protein-based formula compared with those of an extensively hydrolyzed cow’s milk protein-based formula are now available [61], existing recommendations favor the use of the latter, in children with IgE-mediated CMA (one of the reasons being its almost worldwide availability).

### Soy formula vs. extensively hydrolyzed rice formula

Currently, no extensive data are available.

### Partially hydrolyzed formula

The American Academy of Pediatrics defines partially hydrolyzed formulas as those containing reduced proportion of peptides with a molecular weight greater than 5,000 Da [21]. These formulas should not be used for the treatment of suspected or proven CMA or for the diagnostic exclusion diet.

### Other milks

Preparations based on unmodified milk of milks from other mammalian species (sheep, buffalo, horse, camel, or goat milk) or unmodified soy or rice milk should not be used to treat CMA because of their high rate of possible allergenic cross-reactivity and insufficient nutritional value [18]. Similarly, “milk beverages,” derived from almond, coconut, hazelnut, oat, potato, rice, or soya, are not recommended because of their nutritional inadequacy. Compared with cow’s milk, most of them are low in energy and extremely low in protein [36].

## Need for calcium

Calcium supplementation (also phosphorus and vitamin D) is not generally necessary in infants ingesting sufficient amounts of special formula. Whenever milk intake is below 500 ml, assessment by a pediatric dietitian is recommended, and Ca supplement may be needed [36].

## Growth and nutritional concerns

Cow’s milk exclusion diets without appropriate substitution may lead to nutritional deficiencies and poor growth [38]. Discomfort from the underlying illness such as atopic dermatitis or feeding difficulties due to esophageal dysmotility in eosinophilic esophagitis may further contribute to inadequate nutrient intake. Considering these concerns, scientific organizations recommend the use of an age-appropriate milk substitute in children younger than 2 years of age and food counseling. Isolauri et al. analyzed 100 infants with a mean age of 7 months with a diagnosis of atopic dermatitis and challenge-proven CMA who were evaluated for growth during the therapeutic elimination diet [26]. Although clinical control of symptoms was achieved in all patients, mean length SD score and weight-for-length index of patients decreased compared with those of healthy age-matched children, *p* < 0.0001 and *p* = 0.03, respectively. In addition, low serum albumin was seen in 6 %, abnormal urea concentration in 24 %, and low serum phospholipid docosahexaenoic acid in 8 %. The delay in growth was more pronounced in a subgroup of patients with early onset than in those with later of symptoms.

## Duration of milk exclusion diet

Once an infant is diagnosed as having CMA and is placed on an exclusion diet, reevaluation needs to be performed every 6 months if the child is under 1 year of age and every 6–12 months from 1 year of age onward, to determine if the child is a candidate for reintroducing cow’s milk. The BSACI has suggested an escalation of products, a so-called “milk ladder,” starting with baked milk products, as thermal processing reduces allergenicity [36]. If well tolerated, more allergenic products can be reintroduced progressively leaving for the end uncooked cheese and fresh cow’s milk, which should only be introduced in children with demonstrated full tolerance to baked milk products.

## Introduction of complementary food

The earlier recommendations of avoidance or delayed introduction of potentially allergenic foods have been replaced by guidelines recommending exactly the opposite. Several prospective birth cohort studies such as GINI [17], LISA [65], KOALA [57], and Generation R [59] indicated no obvious effect of the delayed introduction of solid foods on the prevalence of food allergies. More recently, a population-based, cross-sectional study, which involved 2,589 infants, found that, regardless of eczema status, delayed dietary introduction of egg was associated with a higher risk of egg allergy [32]. Cooked eggs (i.e., boiled, scrambled, fried, or poached) rather than baked eggs (egg-containing products such as cakes or biscuits) at 4 to 6 months of age were more effective in preventing the development of egg allergy at 1 year of age. This finding would point out to the importance of the way that a food item is prepared in addition to the time at which it is introduced.

At present, there is a lack of convincing scientific evidence indicating that delayed introduction of potentially allergenic foods (e.g., cow’s milk protein [except for whole cow’s milk], eggs, peanuts, tree nuts, fish, and seafood) beyond 4–6 months reduces allergies in infants considered to be at increased risk for the development of allergic diseases. Highly allergenic foods are best first introduced at home, rather than at a day care center or at a restaurant [20].

## Probiotics

The WAO recently concluded that, as of today, no single probiotic supplement or combination of them has shown to dramatically influence the course of allergic manifestations or long-term outcome in a permanent way [19]. One randomized controlled trial (RCT) published subsequently to the WAO document found that the addition of *Lactobacillus rhamnosus* GG (LGG) to the therapeutic formula has an impact on acquisition of tolerance. In this trial particpants were randomly assigned to receive one of the following formulas: extensively hydrolyzed casein, extensively hydrolyzed casein with LGG, hydrolyzed rice, soy, or amino acid-based [4]. The rate of oral tolerance after 1-year treatment determined by food challenge was significantly higher in the groups that received extensively hydrolyzed casein formula whether it was with LGG (78.9 %) or without (43.6 %) compared with the other groups: hydrolyzed rice formula (32.6 %), soy formula (23.6 %), and amino acid-based formula (18.2 %) Repeat studies are needed.

## Induction of oral tolerance

At present, there are no established guidelines or protocols on how to proceed with this aspect of treatment. Once an elimination diet is in place and the patient improves, the next major challenge is the induction of tolerance. Reasoning behind the use of the oral route is to expose the immune system to either low doses of antigen or to antigenically modified molecules, capable of inducing a response of immunotolerance without one of allergy. As shown in two meta-analyses, compared to an elimination diet alone, oral immunotherapy for IgE-mediated CMA showed improved chances of achieving CM’s tolerance [8, 64]. However, those two meta-analyses also showed that development of long-term tolerance was unlikely. Oral immunotherapy, however, poses the risk of severe adverse reactions. Experience, however, indicates that when reactions occur, these are generally mild and short lasting. A possible form of oral immunotherapy could be the use of extensively heated milk as well as egg protein because studies have shown that the protein treated in such manner may be tolerated by children who react to raw cow milk [30, 35]. Although studies are still limited, experts have suggested that an oral challenge under professional supervision using heated milk could be tried in children with CMA. Guidelines still do not recommend the use of baked milk products for desensitization in routine clinical practice.

There is a potential role for probiotics in inducing immunotolerance. A study of the effect of certain probiotic strains on tolerance acquisition in children with CMA gave negative results [24]. However, Berni-Canani et al. [3] randomly allocated infants with CMA while still receiving intact protein formula to a group that received either extensively hydrolyzed casein formula or the same EHCF containing *Lactobacillus* GG. After 6 months of an exclusion diet, a double-blind placebo-controlled milk challenge was performed in 55 patients, and evidence of tolerance was seen in 21.4 and 59.3 %, respectively. The difference in acquisition of immunotolerance was significant only for those children with non-IgE-mediated CMA (*p* = 0.017).

## Prevention of cow’s milk allergy

It could be hypothesized that allergen avoidance during the first few months of life, period of immune immaturity, could be beneficial in allergy prevention. However, evidence points otherwise.

### Diet during pregnancy or lactation

The available data do not support cow’s milk antigen avoidance, and therefore, specific allergen avoidance is not recommended during pregnancy [20, 53]. Still under investigation is whether that recommendation also applies to peanut [37, 55]. The negative impact of dietary restrictions on the nutrition of the pregnant woman and her fetus also needs to be considered when eliminating ubiquitous nutrients.

### Breastfeeding

The mechanisms by which exclusive breastfeeding may help in the prevention of allergic disease are passive and active: passive, by decreasing exposure to exogenous antigens, and active, by providing substances present in breast milk capable of protecting the infant against infections, inducing maturation of the gastrointestinal mucosa, promoting the development of healthy gut microbiota, and conferring immunomodulatory and anti-inflammatory benefits [52].

Although the idea that breast milk is effective for allergy prevention is very logical, scientific evidence demonstrating such beneficial effects is not always supportive [15]. Many factors play a role in making such demonstration difficult, reflecting a variety of methodological problems related with investigating breastfeeding in studies. These include inability to randomize and blind, the retrospective design of many studies and the potential for parental recall bias, imprecise definitions of the intervention with no clear distinction between “exclusive breastfeeding” and “any breastfeeding,” the lack of strict diagnostic criteria for allergic diseases, and, finally, reverse causation, meaning that mothers of infants who show evidence of allergy may continue to breastfeed longer to prevent worsening of symptoms. The Despite the controversy, exclusive breastfeeding for at least 4 months, but preferentially up to 6 months is recommended [15, 21, 52].

### Dietary products with reduced allergenicity

In the following section, we discuss options for those infants who are not going to be breastfed or in whom breastfeeding will be supplemented with formula.

### Hydrolyzed formula

A summary article of reviews and a systematic review of subsequently published trials reported that certain extensively hydrolyzed casein formulas and certain partially hydrolyzed whey formulas are capable of reducing the risk of allergy in high-risk infants [60]. Thus, in high-risk infants who are not being breastfed, hydrolysates of documented safety and efficacy have an indication for infant feeding up to the age of 4 to 6 months. Current recommendations also agree that infants with a documented hereditary risk of allergy (i.e., an affected parent and/or sibling) who are not exclusively breastfed would also benefit from such formulas as a means of preventing allergic reactions, primarily atopic dermatitis [7, 16]. There are no data regarding allergy prevention by special infant formulas in the not-at-risk population.

### Soy protein formula

Compared with cow’s milk formula, soy formula failed to prevent allergy in later infancy and childhood in infants at high risk of allergy who were not completely breastfed, as shown in one meta-analysis of three RCTs [43]. Therefore, soy protein formula has no role for the prevention of allergic diseases [6, 14].

### Amino-acid-based formula

There are no studies using amino-acid-based formulas for allergy prevention.

### Probiotics and/or prebiotics

Several recent meta-analyses have suggested that certain probiotics administered both prenatally and postnatally are effective in preventing eczema [5, 35, 45]. An important limitation of such studies, however, is that all of them pooled data from studies in which different probiotic strains were used, lacking subanalyses to determine effects of individual probiotic strain(s). The World Allergy Organization has concluded that in view of the existing information, probiotics do not have a proven role in the prevention of allergy [19]. The evidence supporting prebiotics and synbiotics positively affecting the development and severity of allergic disease is even weaker [22, 33, 44].

### Long-chain polyunsaturated fatty acids

The balance between pro-inflammatory *n*-6 long-chain polyunsaturated fatty acid (LCPUFA) and anti-inflammatory *n*-3 LCPUFA may play a role in the development of allergy. Epidemiological data support the knowledge that low consumption of oily fish rich in *n*-3 LCPUFA favors the presence of more *n*-6 LCPUFA and contributes to the development of allergy and asthma [23, 42]. However, a 2008 meta-analysis of ten publications (representing six RCTs) found no clear evidence of a benefit of *n*-3 or *n*-6 supplementation for reduction of the risk of allergic sensitization or developing a favorable immunological profile [2]. However, the impact of LCPUFA supplementation may only have a window. Studies suggest that the timing of the intervention may play an important role. The Docosahexaenoic Acid to Optimise Mother Infant Outcome (DOMInO) RCT found that maternal *n*-3 LCPUFA supplementation (900 mg/day) during pregnancy reduced the risk of atopic eczema and egg sensitization during the first year of life but not the overall incidence of IgE-associated allergies [46].

Postnatal supplementation with *n*-3 LCPUFA has shown mixed results: One study suggested a transient effect on symptoms of respiratory disease [39], while another showed no effect [11].

### Other nutritional interventions

Supportive evidence is weak with respect to supplementation with vitamins A, D, and E; zinc; fruit and vegetables; and a Mediterranean diet for the prevention of atopic disease, namely, asthma as concluded by a recent systematic review and meta-analysis of observational trials (no RCTs were identified) [41]. At present, no specific recommendations exist for the doses and timing of these products for allergy prevention.

## Management of anaphylaxis

One recent systematic review found no robust studies investigating the effectiveness of adrenaline (epinephrine), H1 anti-histamines, systemic glucocorticosteroids, or methylxanthines in the management of anaphylaxis [12].

Even if the evidence is limited, the first-line treatment for anaphylaxis is epinephrine (both in the outpatient setting [autoinjector] and in a hospital setting). Other medications used in the management of anaphylaxis include anti-histamines or anti-inflammatory drugs (systemic or topical steroids) [7]. The latter are the main therapy in cases of eosinophilic esophagitis or gastroenteritis in which dietary restriction was not feasible or had failed to improve the disease [48].

## What it is that is not in the guidelines

Infants suspected of having CMA who are not breastfed are placed on a special infant formula for up to 6 weeks (depending on the symptoms) and then challenged. If they do not relapse, they are considered to be either cured or that the diagnosis was incorrect. However, CMA may relapse with symptoms that are different from those seen at presentation. One form of CMA is enteritis which may lead to nutrient malabsorption. It is recommended that infants be followed closely for growth parameters following reintroduction of cow’s milk to their diet.

Another caveat is that infants with CMA may have delayed gastric emptying and present with vomiting hours after having ingested milk or food. In evaluating infants experiencing vomiting and considering gastroesophageal reflux, it has to be kept in mind that in simple gastroesophageal reflux, vomiting occurs during or immediately after a meal (30 min), while vomiting that occurs hours after a meal may be associated to allergy. Ravelli et al. described that in sensitized infants, cow’s milk induces severe gastric dysrhythmia and delayed gastric emptying, which, in turn, may exacerbate GER and induce reflex vomiting [47].

## Summary

In this article, we review current recommendations regarding CMA, which is the most common food allergy in infants and young children. A medical history including history of allergy in close relatives, physical examination, and diagnostic elimination diets are the first steps for the accurate diagnosis and management of these patients. sIgE measurements, SPTs, and oral food challenges are usually performed to determine if the problem is IgE-mediated or not. Strict avoidance of the offending allergen is the only therapeutic option. Recommendations for the primary prevention of allergy include exclusive breastfeeding for at least 4 months and up to 6 months if possible. Infants with a documented hereditary risk of allergy (i.e., an affected parent and/or sibling) who cannot be exclusively breastfed should receive a formula with confirmed reduced allergenicity, i.e., a partially or extensively hydrolyzed as a way to minimize the risk of allergic reactions, primarily atopic dermatitis. There is no evidence that avoidance or delayed introduction of solid foods beyond 4–6 months has a positive effect for allergy prevention.

## Abbreviations

CMA: Cow’s milk allergy
RCT: Randomized controlled trials
sIgE: Specific IgE
SPTs: Skin prick tests
